# Sterol-resistant SCAP Overexpression in Vascular Smooth Muscle Cells Accelerates Atherosclerosis by Increasing Local Vascular Inflammation through Activation of the NLRP3 Inflammasome in Mice

**DOI:** 10.14336/AD.2020.1120

**Published:** 2021-06-01

**Authors:** Danyang Li, Mihua Liu, Zhe Li, Guo Zheng, Amei Chen, Lei Zhao, Ping Yang, Li Wei, Yaxi Chen, Xiong Z. Ruan

**Affiliations:** ^1^Centre for Lipid Research & Key Laboratory of Molecular Biology for Infectious Diseases (Ministry of Education), Institute for Viral Hepatitis, Department of Infectious Diseases, the Second Affiliated Hospital, Chongqing Medical University, Chongqing, China.; ^2^National Clinical Research Center for Aging and Medicine, Huashan Hospital, Fudan University, Shanghai, China.; ^3^John Moorhead Research Laboratory, Centre for Nephrology, University College London Medical School, Royal Free Campus, University College London, London, United Kingdom.

**Keywords:** SCAP, Inflammation, Atherosclerosis, VSMC, NLRP3

## Abstract

Atherosclerosis is a serious age-related pathology, and one of its hallmarks is the presence of chronic inflammation. Sterol regulatory element-binding protein (SREBP) cleavage-activating protein (SCAP) is a cholesterol sensor that plays an essential role in regulating intracellular cholesterol homeostasis. Accordingly, dysregulation of the SCAP-SREBP pathway has been reported to be closely associated with an increased risk of obesity, hypercholesterolemia, and cardiovascular disease. In this study, we explored whether sterol-resistant SCAP (D443N mutation) in vascular smooth muscle cells (VSMCs) of mice promotes vascular inflammation and accelerates the occurrence and progression of atherosclerosis. We established a transgenic knock-in mouse model of atherosclerosis with an activating D443N mutation at the sterol-sensing domain of SCAP (SCAP^D443N^) by microinjection. Next, SCAP^D443N^/ApoE^-/-^ mice were generated by crossing SCAP^D443N^ mice with apolipoprotein E^-/-^ (ApoE^-/-^) background mice. We found that sterol-resistant SCAP markedly amplified and accelerated the progression of atherosclerotic plaques in SCAP^D443N^/ApoE^-/-^ mice compared with that in control ApoE^-/-^ mice. Similarly, in SCAP^D443N^ mice, aortic atherosclerotic plaques both appeared earlier and were greater in number than that in control SCAP^+/+^ mice, both of which were fed a Western diet for 12 or 24 weeks. Moreover, we observed that sterol-resistant SCAP significantly increased local inflammation and induced endothelial dysfunction in the aortas of SCAP^D443N^ mice and SCAP^D443N^/ApoE^-/-^ mice. *In vitro*, we also found that sterol-resistant SCAP overexpression in VSMCs increased the release of inflammatory cytokines and induced endothelial cell injury when both cell types were cocultured. Furthermore, we demonstrated that sterol-resistant SCAP overexpression in VSMCs promoted SCAP and NLRP3 inflammasome cotranslocation to the Golgi and increased the activation of the NLRP3 inflammasome pathway. These findings suggested that sterol-resistant SCAP in VSMCs of mice induced vascular inflammation and endothelial dysfunction, consequently accelerating atherosclerosis by activating the NLRP3 inflammasome pathway.

Aging is one of the main risk factors for the development of cardiovascular disease. Sterol regulatory element-binding protein (SREBP) cleavage-activating protein (SCAP) is progressively increased during the aging process [1]. SREBPs are key transcription factors that regulate a series of genes involved in maintaining intracellular cholesterol homeostasis [2]. SCAP acts as an escort chaperone for SREBPs, and the retention of the SCAP-SREBP complex in the endoplasmic reticulum (ER) is mediated by the binding of SCAP to insulin-induced gene (INSIG) proteins. When sterol levels fall, SCAP dissociates from INSIGs and transports SREBPs from the ER to the Golgi apparatus for subsequent activation to release the nuclear SREBPs. When the sterol level is sufficient, SCAP can bind to INSIGs, preventing the SCAP-SREBP complex from exiting the ER, thereby reducing the activation of SREBP cleavage. Therefore, sterol-induced binding of SCAP to INSIGs is a crucial event in sterol-mediated negative feedback regulation in mammalian cells. However, the dysregulation of the SCAP-SREBP pathway in the development of atherosclerosis is not fully clarified and is still being enthusiastically explored.

Until now, several SCAP inhibitors have been reported, such as fatostatin [3, 4], betulin [5], 25-hydroxyvitamin D [6] and lycorine [7]. Some of these inhibitors are already in preclinical [8] and clinical studies [9]. These inhibitors block the transport of the SCAP/SREBP complex to the Golgi, inhibiting SREBP activation and alleviating hyperlipidemia [5, 7]. Fatostatin [10] and betulin [5] inhibit lipid metabolism by binding to SCAP, inhibiting the transport of SCAP-SREBPs to the Golgi and inhibiting SREBP maturation. Distinct from sterols and current SCAP inhibitors, 25-hydroxyvitamin D regulates lipid metabolism by inducing proteolytic cleavage of SCAP, leading to its proteasomal degradation [6]. Similarly, lycorine induces SCAP degradation in lysosomes independent of the ubiquitin-proteasome pathway. Lycorine improves high-fat diet-induced insulin resistance, dyslipidemia, and obesity in mice [7]. Therefore, inhibition of SCAP signaling seems to be an attractive strategy to treat hypercholesterolemia and metabolic diseases.

Sterol-mediated negative feedback regulation of SREBP cleavage is dramatically impaired by SCAP mutants [11]. Accumulating evidence has highlighted that the Y298C, L315F or V439G mutation in SCAP is resistant to the suppressive action of sterol and abolishes SCAP-INSIG interactions, facilitating constitutive ER exit and SREBP2 activation [11-13]. However, the D428A, Y234A or K305R mutation in SCAP fails to dissociate from INSIGs and inhibits the transport of the SCAP-SREBP complex to the Golgi, thus preventing SREBP activation even under conditions of sterol depletion [14-16]. Thus, SCAP is an indispensable protein for the migration of the SCAP-SREBP complex from the ER to the Golgi and activation of SREBPs [17]. Because of a guanine to adenine substitution, a D443N point mutation is generated in the sterol-sensitive domain of SCAP, leading to the sterol resistance of SCAP (D443N). Sterol-resistant SCAP (D443N) causes insensitivity to sterol and facilitates SCAP-SREBP complex translocation from the ER to the Golgi and proteolytic processing of native SREBPs in the Golgi, even in the presence of sterols [18, 19]. Although these mutations indicated that increased SCAP signaling would promote hypercholesterolemia, the role of sterol-resistant SCAP in atherosclerosis has not been studied. Additionally, the D443N mutation in SCAP is a highly specific event that endows SCAP with the ability to resist sterol-mediated feedback regulation. To elucidate the mechanisms of sterol-resistant SCAP in the development of atherosclerosis, it is crucial to generate an improved mouse model of atherosclerosis with an activating D443N mutation in SCAP.

Chronic low-grade inflammation is a characteristic feature of both aging and atherosclerosis [20]. SREBPs also play a role in aging by inducing inflammatory responses [21]. SREBP-1 and the inflammatory response were clearly increased in atherosclerotic artery specimens from patients with diabetes [22]. Our group demonstrated that crosstalk between the TLR4-MyD88-NF-κB and SCAP-SREBP2 pathways mediates macrophage foam cell formation [23]. Additionally, atheroprone flow induces the nod-like receptor protein-3 (NLRP3) inflammasome in the endothelium through SREBP2, and the activated NLRP3 inflammasome synergizes with hyperlipidemia to increase atherosclerosis in apolipoprotein E^-/-^ (ApoE^-/-^) mice [24]. The NLRP3 inflammasome has recently been discovered as a major driver of inflammation, thus accelerating aging and atherosclerosis [25]. NLRP3 inflammasomes are strongly expressed in the aortas of patients with coronary atherosclerosis [26], and NLRP3 ablation mitigates the development of atherosclerosis in mice [27]. Notably, the NLRP3 inflammasome triggers the maturation and production of proinflammatory cytokines, such as interleukin-1β (IL-1β) and IL-18. Indeed, IL-1β and IL-18 were markedly increased in patients with coronary artery disease [28]. By contrast, the genetic deletion of IL-1β and IL-18 significantly reduced the development of atherosclerosis in ApoE^-/-^ mice [29, 30]. However, the underlying mechanism by which the cholesterol homeostatic regulator SCAP-SREBP2 mediates NLRP3 in?ammasome activation in vascular inflammation and atherosclerosis is unclear. Hence, we explored the role of sterol-resistant SCAP in NLRP3 inflammasome activation and subsequent sterile inflammation.

In this study, we created a new model of VSMCs specifically expressing sterol-resistant SCAP (D443N mutant) in both C57BL/6J and ApoE^-/-^ mice to reveal the effects of SCAP on the NLRP3 inflammasome in VSMCs and the development of atherosclerosis. This work revealed, for the first time, the molecular mechanisms by which sterol-resistant SCAP interacts with the inflammasome pathway, promoting endothelial dysfunction and atherosclerotic lesion development.

## MATERIALS AND METHODS

### Mice

The SM22 promoter (pSM22) was amplified from the genomic DNA of mice by nested PCR, and the SCAP (D443N) mutant amplified from the plasmid pTK-HSV-SCAP (D443N) was cloned into the pGL3-control vector to construct pGL3-SM22-SCAP (D443N). Next, pGL3-SM22-SCAP (D443N) was prokaryotically expressed and microinjected to generate SM22 promoter-driven sterol-resistant SCAP (D443N) overexpression (SCAP^D443N^) mice (Nanjing Model Animal Centre, China).

SCAP^D443N^/ApoE^-/-^ mice were created by crossing ApoE^-/-^ mice (The Jackson Laboratory, Bar Harbor, ME, USA) with SCAP^D443N^ mice. Eight-week-old male ApoE^-/-^ mice and SCAP^D443N^/ApoE^-/-^ mice were fed a Western diet (D12079B; New Brunswick, NJ, USA) for 12 weeks. Eight-week-old male wild-type mice and SCAP^D443N^ mice were fed a Western diet for 12 or 24 weeks. No significant difference was found in the survival and body weight between the two mouse groups. The animals were euthanized under deep anesthesia by intraperitoneal injection with an overdose of pentobarbital sodium (200 mg/kg) to obtain their samples. All the animal experimental procedures conformed to the Guide for the Care and Use of Laboratory Animals published by the US National Institutes of Health (National Research Council, 8th Edition, 2011) and regulations issued by the Animal Ethics Committee of Chongqing Medical University.

### Cells

Human vascular smooth muscle cells (VSMCs) were purchased from American Type Culture Collection (Manassas, VA, USA). VSMCs were cultured in F12 medium (HyClone, Logan, Utah, USA) supplemented with 10% fetal bovine serum (HyClone, Logan, Utah, USA), 100 U/mL of penicillin-streptomycin and insulin-transferrin-selenium (Gibco, Thermo Fisher Scientific, Eugene, Oregon, USA). After VSMCs were grown to 70-80% confluence, the cells were cultured with serum-free F12 medium for 24 h, and the conditioned medium was collected and centrifuged for 10 min at 1000 r/min. The supernatants were collected and filtered through a 0.22-μm pore size filter and were used as the VSMC-conditioned medium[31]. SCAP-overexpressing VSMC-conditioned medium was collected from SCAP-overexpressing VSMCs after 4 h of treatment with or without 20 μM lycorine. Human umbilical vein endothelial cells (HUVECs) were cultured in Roswell Park Memorial Institute (RPMI) 1640 medium (HyClone, Logan, Utah, USA) supplemented with 10% fetal bovine serum and 100 U/mL of penicillin-streptomycin. HUVECs were seeded at a density of 1×10^6^ cells/mL. After the cells were grown to confluence and became quiescent, they were incubated with VSMC-conditioned medium and 100 μg/ml of LDL for 24 h..

**Table 1:** qRT-PCR primer sequences used in this study.

Gene names	Organism	Forward	Reverse
β-actin	Mouse	TGCCCTGAGGCTCTTTTCC	TCGTGGATGCCACAGGATT
β-actin	Human	CCTGGCACCCAGCACAAT	GCCGATCCACACGGAGTA
CCL-5	Mouse	GCTGCTTTGCCTACCTCTCC	TCGAGTGACAAACACGACTGC
CX3CL1	Mouse	AAATGCGAAATCATGTGCGAC	CCTGGTTTAGCTGATAGCGGAT
IL-1β	Mouse	GAAATGCCACCTTTTGACAGTG	TGGATGCTCTCATCAGGACAG
IL-1β	Human	ATGATGGCTTATTACAGTGGCAA	GTCGGAGATTCGTAGCTGGA
IL-18	Mouse	GACTCTTGCGTCAACTTCAAGG	CAGGCTGTCTTTTGTCAACGA
IL-18	Human	TCTTCATTGACCAAGGAAATCGG	TCCGGGGTGCATTATCTCTAC
VCAM-1	Mouse	AGTTGGGGATTCGGTTGTTCT	CCCCTCATTCCTTACCACCC
VCAM-1	Human	GGGAAGATGGTCGTGATCCTT	TCTGGGGTGGTCTCGATTTTA
ICAM-1	Mouse	GTGATGCTCAGGTATCCATCCA	CACAGTTCTCAAAGCACAGCG
ICAM-1	Human	TTGGGCATAGAGACCCCGTT	GCACATTGCTCAGTTCATACACC
MCP-1	Mouse	GTCTGTGCTGACCCCAAGAAG	TGGTTCCGATCCAGGTTTTTA
MIP-1α	Mouse	TGTACCATGACACTCTGCAAC	CAACGATGAATTGGCGTGGAA
MIP-1β	Mouse	TTCCTGCTGTTTCTCTTACACCT	CTGTCTGCCTCTTTTGGTCAG
TNF-α	Mouse	GGAGAAGGGTGACCGACTCA	TGCCCAGACTCGGCAAAG
TNF-β	Mouse	ATGACACCACCTGAACGTCTCTTC	CTACAGAGCGAAGGCTCCAAAGAAGACAGTACT
SCAP	Human	GGGAACTTCTGGCAGAATGAC	CTGGTGGATGGTCCCAATG

### Lentivirus infection

Lentivirus containing the human full-length cDNA of SCAP (LV-SCAP) was purchased from GeneCopoeia (GuangZhou, China). The lentiviruses were transfected into VSMCs at an MOI of 50 [32]. PCR and Western blot analysis were used to verified the efficiency of the lentivirus-based vectors. Lenti-NC expressing EGFP was used as the negative control.

### Quantitative real-time polymerase chain reaction (qRT-PCR)

Total RNA of the tissues and VSMCs was extracted by TRIzol reagent (Thermo Fisher Scientific). Subsequently, PrimeScript RT Master Mix (Takara) was used to reverse transcribe total RNA to first-strand cDNA. Next, quantitative RT-PCR was performed using the SYBR PrimeScript RT-PCR kit (Takara). The relative gene expression was calculated by normalizing to β-actin expression as the internal control. The primer sequences are shown in Table 1.

### IL-1β and IL-18 secretion assay

Following treatment, the cell culture supernatants in each group were collected and centrifuged for 10 min at 1000 rpm. The concentrations of IL-1β and IL-18 in VSMC supernatants were measured using an enzyme-linked immunosorbent assay (ELISA) kit (Enzyme-linked Biotechnology, Shanghai, China).

### Oil Red O staining, Masson’s trichrome staining and Sirius red staining

Frozen slices from euthanized mice were permeabilized with 4% paraformaldehyde for 20 min. After rinsing with PBS for 5 min for approximately 3 times, the slices were stained with Oil Red O for 15 min at 37°C. Next, the slices were washed with ddH_2_O and counterstained with hematoxylin for 8 min. Sirius red staining and Masson’s trichrome staining were performed according to the manufacturer’s instructions (Solarbio Life Science, Beijing, China). Finally, all the slides were examined under a light microscope.

### Lipid analyses

Serum samples were collected from anesthetized mice through cardiac puncture, and the levels of total cholesterol (TC), total triglycerides (TG), low-density lipoprotein (LDL) cholesterol, and high-density lipoprotein (HDL) cholesterol were measured by enzymatic methods (Nanjing JianCheng, Nanjing, China).

### Coimmunoprecipitation and Western blotting

Cell lysates were incubated with IgG or anti-SCAP antibody for 2?h at 4?°C, and then magnetic beads were bound to the SCAP immune complexes at 4°C overnight. After incubation, the magnetic beads were washed with phosphate-buffered saline; the proteins were collected from the magnetic beads, mixed with protein loading buffer, and boiled for 8 min at 100°C. Finally, the proteins were detected by Western blotting analysis.

The proteins were separated by SDS-PAGE and then transferred to a PVDF membrane. After that, the membranes were blocked with 3% bovine serum albumin for 2 h, followed by incubation with the following primary antibodies: anti-nSREBP2 (ab30682; Abcam), anti-caspase 1 (22915-1-AP; Proteintech), anti-cleaved caspase-1 (4199S, Cell Signaling Technology), anti-IL-1β (A11370; Abclonal), anti-IL-18 (10663-1-AP; Proteintech), anti-VCAM-1 (11444-1-AP; Proteintech), anti-ICAM-1 (10020-1-AP; Proteintech) and anti-NLRP3 (NBP1-97601; Novus Biologicals). After overnight incubation at 4°C, the membranes were incubated with horseradish peroxidase-labeled goat anti-rabbit with rotation for 2 h at 37°C. Finally, detection was performed using an ECL chemical luminescent detection kit (Bio-Rad), and the bands were further analyzed using Quantity One software. The expression of the target protein was normalized to β-actin expression.

### Immunohistochemical staining

Frozen slices were fixed with 4% paraformaldehyde for 15 min and incubated with 0.3% Triton X-100 for 15 min. The slices were incubated with 3% hydrogen peroxide for 10 min, and nonspecific epitopes were blocked with goat serum for 15 min. Each slice was incubated with the following primary antibodies: anti-IL-1β (A11370; Abclonal), anti-IL-18 (10663-1-AP; Proteintech), anti-VCAM-1 (11444-1-AP; Proteintech), anti-ICAM-1 (10020-1-AP; Proteintech), anti-IL-6 (66146-1-1g; Proteintech), anti-TNF-α (A11534; Abclonal), and anti-CD68 (25747-1-AP; Proteintech). After overnight incubation, the slices were incubated with Streptomyces anti-biotin peroxidase solution for 1 h at 37 ?. The slices were then visualized using DAB solution (Zhongshanjingqiao Biotechnical) and counterstained with hematoxylin for 8 min. Finally, all the slides were examined under a light microscope, and images were analyzed using Image Pro Plus software.

### Immunofluorescent staining

Frozen slices or cells were fixed with 4% paraformaldehyde for 15 min and incubated with 0.3% Triton X100 for 15 min. After blocking with 3% bovine serum albumin, the slices were incubated with the following primary antibodies: anti-SCAP (NBP2-04113; Novus Biologicals), anti-Golgi (A-21270; Thermo Fisher), anti-α-SMA (55135-1-AP; Proteintech), anti-NLRP3, anti-VCAM-1, and anti-ICAM-1. After overnight incubation, the slices or cells were then incubated with fluorescence-conjugated secondary antibodies for 1 h. Finally, the slices or cells were incubated with Hoechst for 5 min, and then images were captured under a Zeiss fluorescence microscope and analyzed using Image Pro Plus software.

**Figure 1.**
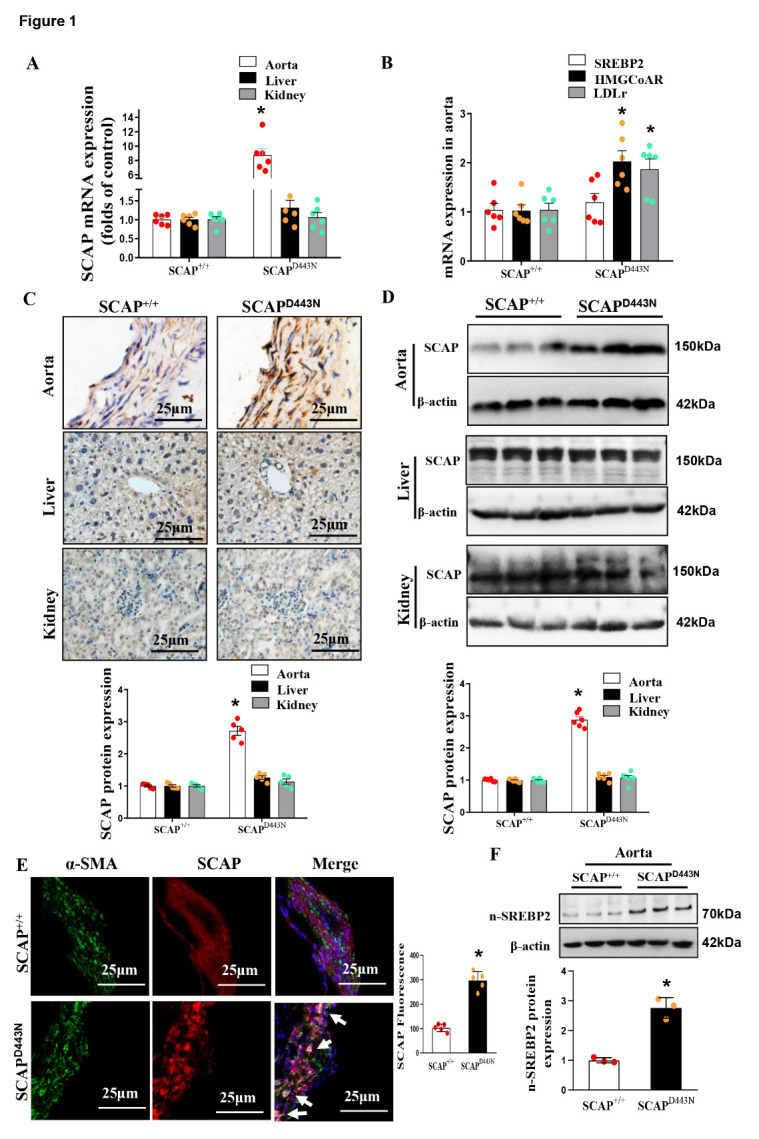
Evaluation of the SM22 promoter-driven sterol-resistant SCAP (D443N mutant) transgenic mouse model. (A) mRNA expression of SCAP in the aorta, liver and kidney as measured by qRT-PCR. (B) mRNA expression of SREBP2, HMGCoAR, LDLr in the aorta as measured by qRT-PCR. (C) Expression of SCAP in the aorta, liver and kidney as determined by immune-histochemical staining. (D) Representative immunoblots of SCAP protein expression in the aorta, liver and kidney. (E) Double immune?uorescence staining for SCAP and α-SMA in the aortas of SCAP^D443N^ mice and SCAP^+/+^ mice. (F) Representative immunoblots of n-SREBP2 protein expression in the aorta of SCAP^D443N^ mice and SCAP^+/+^ mice. The data are presented as the means±SD of 6 independent experiments. *P<0.05, vs. the SCAP^+/+^ group. Statistical significance was calculated for the biological replicates by 2-tailed Student’s t test.

### Flow cytometry analysis

Following treatment, HUVECs were collected and resuspended in 1 ml of ice-cold PBS at a density of 1×10^6^ cells/mL. The samples were stained with 10 μl of Annexin V-FITC for 15?min in the dark, and then 10 μl of propidium iodide was added and incubated for 10 additional min. Finally, the samples were immediately detected by flow cytometry (BD Biosciences, US) and the data were analyzed using FlowJo software version 10.

### Monocyte adhesion assay

The monocyte adhesion assay was performed as previously described [33]. VSMC-conditioned medium was collected and centrifuged for 10 min at 1000 r/min, and the supernatants were filtered through a 0.22-μm pore size filter. Confluent HUVECs were incubated with VSMC-conditioned medium and 100 μg/ml of LDL for 24 h. Subsequently, HUVECs were washed with PBS and rinsed with medium, and then THP-1 monocytes (1×10^6^ cells/well) were added to each well and incubated with HUVECs at 37°C for 20 min. The nonadherent monocytes were rinsed off, and the wells were fixed with 1% paraformaldehyde for 10 min. The number of attached monocytes was counted.

### Cell migration analysis

VSMC migration was evaluated by wound healing analysis. VSMCs were seeded at a density of 1×10^6^ cells/mL on 6-well cell culture plate. After the cells were grown to 90% confluence, VSMCs were scraped with a 200 μL aseptic pipette. Subsequently, cell debris or floating cells were washed with PBS twice and fresh medium was added. After 24 h, the width of the wound was recorded and analyzed.

### Statistical analysis

The results are expressed as means±SD. The differences in the data between distinct experimental groups were analyzed using 2-tailed Student’s t test. Comparisons between groups were performed using 2-way ANOVA, followed by Q-tests using SPSS 18.0 software. *P*<0.05 was considered statistically significant, and *P*<0.01 was considered statistically remarkably significant.

## RESULTS

### Evaluation of the SM22 promoter-driven sterol-resistant SCAP-overexpressing mouse model

Initially, qRT-PCR revealed a significant increase in the expression of SCAP mRNA in the aorta of SCAP^D443N^ mice but not in the liver and kidney (Fig. 1A). Similarly, we observed that downstream signaling molecules of SCAP, such as 3-hydroxy-3-methyl glutaryl coenzyme A reductase (HMGCoAR) and low-density lipoprotein receptor (LDLr) mRNA, were significantly increased in the aortas of SCAP^D443N^ mice (Fig. 1B). Immunohistochemistry and Western blotting analyses also demonstrated that SCAP expression levels were markedly increased in the aortas of SCAP^D443N^ mice compared with those of wild-type (SCAP^+/+^) mice (Figure 1C-D). To observe the expression of SCAP in VSMCs, we used α-SMA (green) to label the VSMCs and costained for SCAP (red). As expected, the expression of SCAP and α-SMA was significantly increased in the VSMCs media of SCAP^D443N^ mice (Fig. 1E). Western blotting analyses showed that sterol-resistant SCAP enhanced the protein expression of n-SREBP2 in the aortas of SCAP^D443N^ mice (Fig. 1F). Immuno?uorescence staining revealed that the SCAP expression levels were markedly increased in the atherosclerotic lesions of ApoE^-/-^ mice (Supplemental Fig. 1A). These data indicated that the upregulation of SCAP expression occurred in VSMCs from the aorta of SCAP^D443N^ mice.

### Sterol-resistant SCAP overexpression promotes atherosclerotic lesion progression in the aortas of ApoE^-/-^ mice

**Figure 2.**
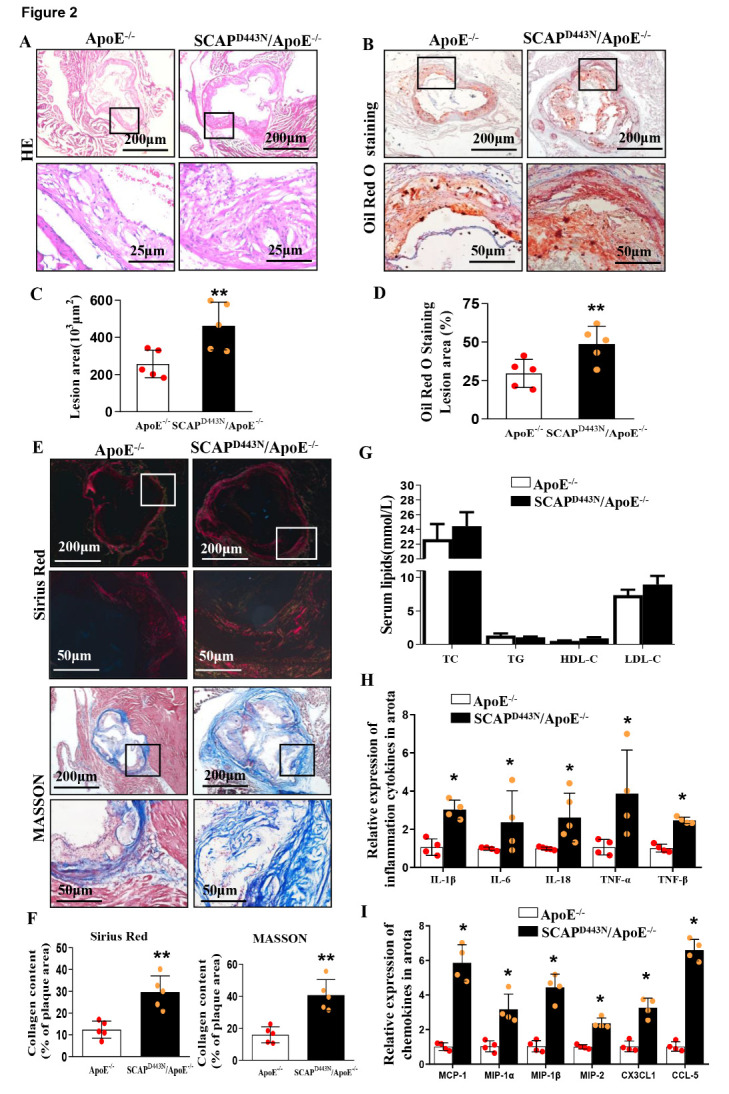
Sterol-resistant SCAP promotes atherosclerotic lesion progression in ApoE^-/-^ mice. (A) Representative images of the aortic root stained with hematoxylin and eosin (H&E). (B) Representative images of the aortic root stained with Oil Red O. (C) Quantification of the lesion cross-sectional area in H&E-stained aortic root sections in (A) (n=5). (D) Quantification of the lesion volume of Oil Red O-stained aortic root sections in (B) (n=5). (E) Representative images of the aortic root stained with Sirius red and Masson’s trichrome stain. (F) Quantification of the content of collagen fibers in the aortic roots (n=5). (G) Serum levels of TC, TG, HDL-C and LDL-C in ApoE^-/-^ mice and SCAP^D443N^/ApoE^-/-^ mice (n=6). The data are presented as the means±SD of 5 independent experiments. **P<0.01, vs. the ApoE^-/-^ group. (H) mRNA expression of proin?ammatory cytokines (IL-1β, IL-6, IL-18, TNF-α and TNF-β) as measured by qRT-PCR (n=4). (I) mRNA expression of pro-chemokine cytokines (MCP-1, MIP-1α, MIP-1β, MIP-2, CX3CL1 and CCL-5) as measured by qRT-PCR (n=4). The data are presented as the means±SEM of 4 independent experiments. *P<0.05, vs. the ApoE^-/-^ group. Statistical significance was calculated for the biological replicates by 2-tailed Student’s t test.

Next, SCAP^D443N^/ApoE^-/-^ mice were generated by crossing SCAP^D443N^ mice with ApoE^-/-^ mice. SCAP^D443N^/ApoE^-/-^ male mice were then fed a Western diet for 12 weeks to evaluate the atherosclerotic plaque burden compared with ApoE^-/-^ mice (control). The data showed significant increases in the sizes of both the plaques (Fig. 2A, C) and Oil Red O-stained lesion area (Fig. 2B, D) in SCAP^D443N^/ApoE^-/-^ mice. To test whether sterol-resistant SCAP influences plaque stability, the collagen contents of the plaque were analyzed by Sirius red and Masson’s trichrome staining. The tissue section results showed that the collagen-positive area was dramatically increased in SCAP^D443N^/ApoE^-/-^ mice compared with that in control mice (Fig. 2E-F). No significant differences were observed in the serum TC, TG, LDL cholesterol, and HDL cholesterol levels between SCAP^D443N^/ApoE^-/-^ mice and ApoE^-/-^ mice (Fig. 2G). These *in vivo* data suggested that sterol-resistant SCAP overexpression in VSMCs promoted the development of atherosclerosis and contributed to plaque stability in ApoE^-/-^ mice.

### An elevated inflammatory response is evident in the atherosclerotic aortic lesions of SCAP^D443N^/ApoE^-/-^ mice

**Figure 3.**
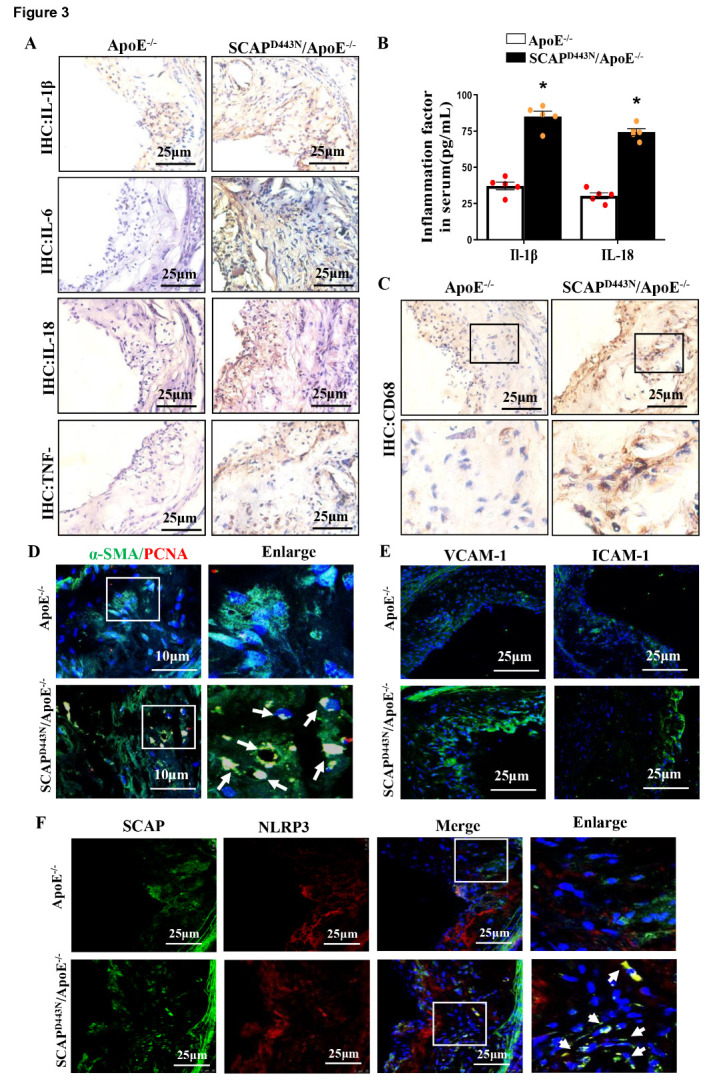
Sterol-resistant SCAP increases inflammation in advanced atherosclerotic progression. (A) Expression of proin?ammatory cytokines (IL-1β, IL-6, IL-18, and TNF-α) in aortas as determined by immunohistochemical staining. (B) Expression of IL-1β and IL-18 in serum as measured by ELISA. (C) Expression of CD68 in aortas as determined by immunohistochemical staining. (D) Double immuno?uorescence staining for α-SMA and PCNA in the aortas of ApoE^-/-^ mice and SCAP^D443N^/ApoE^-/-^ mice. (E) Representative images of immuno?uorescence staining of ICAM-1 and VCAM-1 proteins in the atherosclerotic lesions of ApoE^-/-^ mice and SCAP^D443N^/ApoE^-/-^ mice. (F) Representative images of immuno?uorescence staining of SCAP (green) with NLRP3 (red) in the atherosclerotic lesions of ApoE^-/-^ and SCAP^D443N^/ApoE^-/-^ mice. The data are presented as the means±SD of 5 independent experiments. *P<0.05, vs. the ApoE^-/-^ group. Statistical significance was calculated for the biological replicates by 2-tailed Student’s t test.

However, the mRNA expression of proin?ammatory cytokines and chemokines was significantly increased in the aortas of SCAP^D443N^/ApoE^-/-^ mice (Figure 2H-I). Similarly, the expression levels of tumor necrosis factor-α (TNF-α), IL-1β, IL-6, and IL-18 were increased in the aortas of SCAP^D443N^/ApoE^-/-^ mice (Figure 3A). The production of IL-1β and IL-18 significantly increased in the serum of SCAP^D443N^/ApoE^-/-^ mice (Figure 3B). Furthermore, we found that sterol-resistant SCAP significantly increased macrophage infiltration in atherosclerotic lesions (Figure 3C). Next, we measured the effect of SCAP on VSMC proliferation in the atherosclerotic aortic lesions. We utilized α-SMA to label the VSMCs of aortic plaques and costained with proliferating cell nuclear antigen (PCNA). The data showed that PCNA was markedly upregulated in the VSMCs of SCAP^D443N^/ApoE^-/-^ mice (Figure 3D), suggesting that SCAP overexpression could increase VSMC proliferation in the aortas of ApoE^-/-^ mice. Sterol-resistant SCAP overexpression in VSMCs exhibited a significantly increased migration in wound healing analysis (Supplemental Figure 1B). Immuno?uorescence staining of aortic roots revealed that the expression levels of vascular cell adhesion molecule 1 (VCAM-1) and intercellular adhesion molecule-1 (ICAM-1) were markedly increased in SCAP^D443N^/ApoE^-/-^ mice (Figure 3E). Interestingly, the colocalization of NLRP3 with SCAP was significantly increased in the atherosclerotic lesions of SCAP^D443N^/ApoE^-/-^ mice (Figure 3F). These data suggested that sterol-resistant SCAP overexpression in VSMCs promoted the development of atherosclerosis by upregulating local aortic inflammation independent of the serum cholesterol levels.

**Figure 4.**
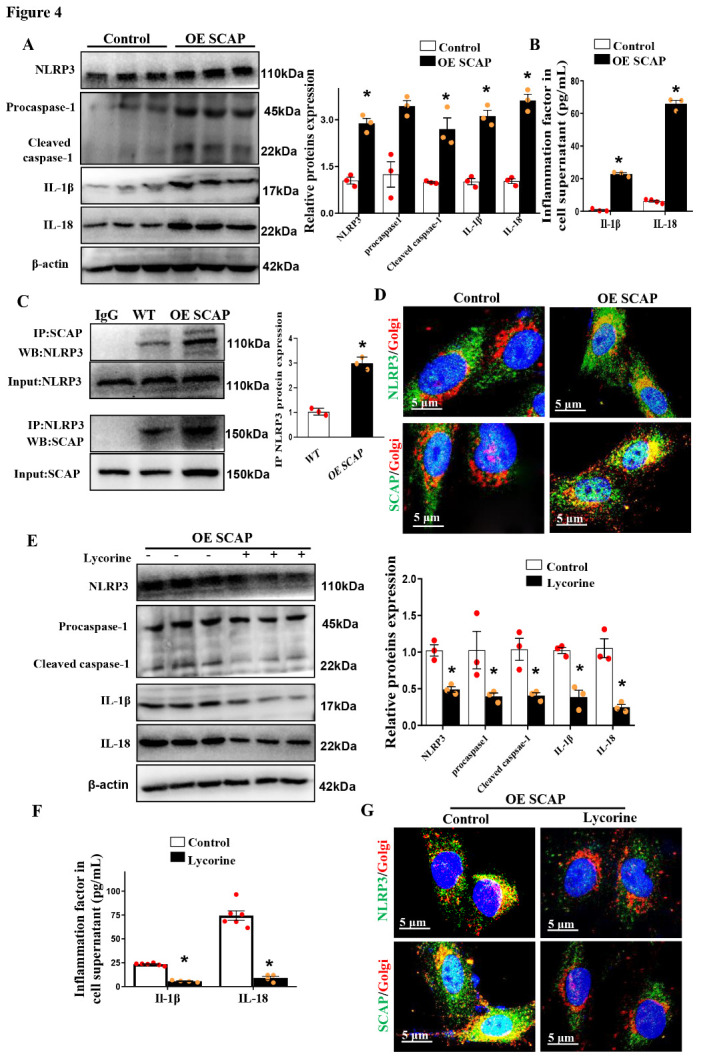
Sterol-resistant SCAP overexpression induces the activation of NLRP3 in?ammasomes in VSMCs. (A) Representative immunoblots for NLRP3, Procaspase-1, cleaved caspase-1, IL-1β, and IL-18 in SCAP-overexpressing VSMCs. (B) Expression of IL-1β and IL-18 in VSMC supernatants as measured by ELISA. (C) Immunoblotting of immunoprecipitation with anti-SCAP or anti-NLRP3 in SCAP-overexpressing VSMCs. (D) Representative images of immuno?uorescence staining of NLRP3 (green) or SCAP (green) with Golgi(red) in control and SCAP-overexpressing VSMCs. (E) Representative immunoblot for NLRP3, Pro-caspase-1, cleaved caspase-1, IL-1β, and IL-18 in SCAP-overexpressing VSMCs treated with lycorine for 4 h. (F) Expression of IL-1β and IL-18 in VSMC supernatants as measured by ELISA. (G) Representative images of the immuno?uorescence staining of NLRP3 (green) or SCAP (green) with Golgi (red) in SCAP VSMCs after treatment with lycorine for 4 h. The data are presented as the means±SD of 3 independent experiments. *P<0.05, vs the control group. Statistical significance was calculated for to the biological replicates by 2-tailed Student’s t test.

### Sterol-resistant SCAP overexpression induces the activation of NLRP3 in?ammasomes in VSMCs

Furthermore, we found that sterol-resistant SCAP overexpression in VSMCs significantly increased the protein expression of in?ammasome components and in?ammatory cytokines, including NLRP3, procaspase-1, cleaved caspase-1, IL-1β, and IL-18 (Fig. 4A). The levels of proin?ammatory cytokines (IL-1β and IL-18) were also markedly upregulated in the SCAP-overexpressing cell culture supernatant (Fig. 4B). Previous studies have demonstrated that SCAP-SREBP2 is associated with NLRP3 to form a ternary complex that translocates from the endoplasmic reticulum to the Golgi apparatus, which is required for optimal NLRP3 inflammasome assembly and activation in macrophages [34, 35]. Our data revealed that NLRP3 was also associated with SCAP in VSMCs, and the correlation between them was markedly enhanced after SCAP overexpression, as demonstrated by coimmunoprecipitation analysis (Figure 4C). Moreover, sterol-resistant SCAP overexpression significantly increased the colocalization of SCAP and NLRP3 with the Golgi in VSMCs (Figure 4D), a finding that agreed with previous study findings in macrophages [35]. Additionally, sterol-resistant SCAP-overexpressing VSMCs were incubated with the SCAP inhibitor lycorine. The expression levels of NLRP3, caspase-1 activation, the production of IL-1β and IL-18 (Figure 4E-F) and the colocalization of SCAP or NLRP3 with the Golgi in VSMCs were significantly inhibited in the presence of lycorine (Figure 4G). These results suggested that sterol-resistant SCAP overexpression in VSMCs promoted SCAP and NLRP3 inflammasome cotranslocation to the Golgi and induced the activation of the NLRP3 inflammasome pathway.

**Figure 5.**
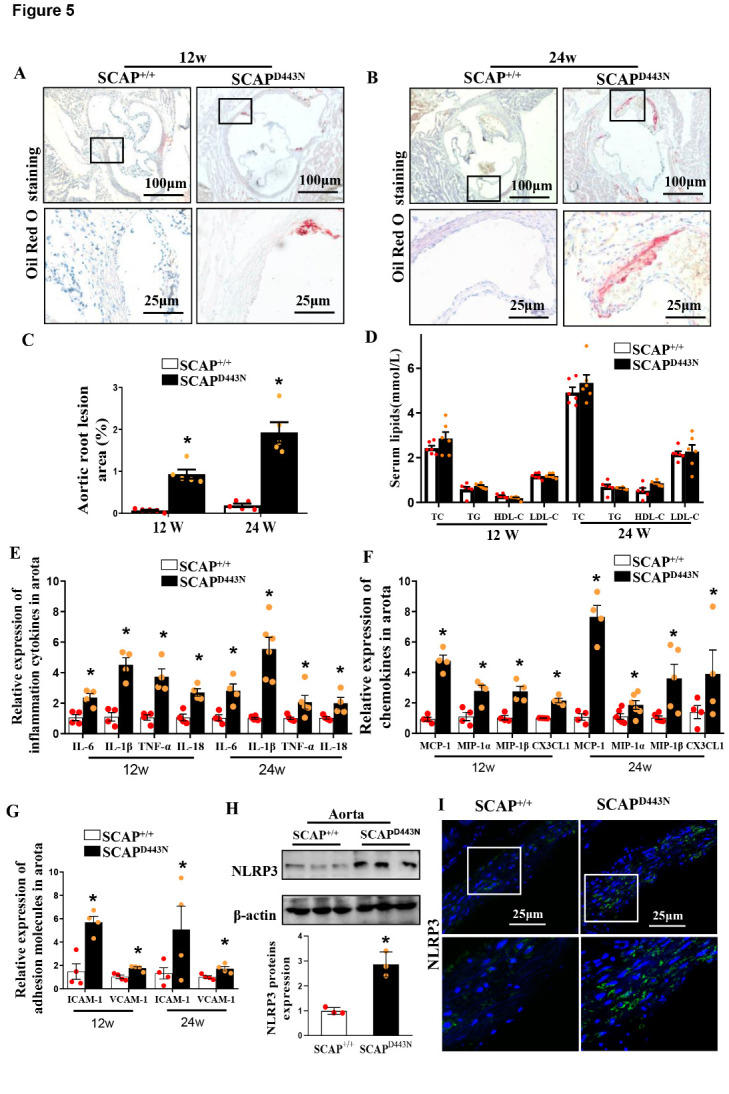
Sterol-resistant SCAP overexpression in VSMCs causes inflammation and lipid deposition in the aortas of SCAP^D443N^ mice. (A and B) Representative images of an aortic root stained with Oil Red O in SCAP^D443N^ mice and SCAP^+/+^ mice after 12 or 24 weeks of Western diet feeding. (C) Quantification of plaque areas in SCAP^D443N^ mice and SCAP^+/+^ mice after 12 or 24 weeks of Western diet feeding (n=5). (D) Plasma levels of TC, TG, LDL-C and HDL-C in SCAP^D443N^ mice and SCAP^+/+^ mice after 12 or 24 weeks of Western diet feeding (n=5). (E) mRNA expression of proin?ammatory cytokines (IL-1β, IL-6, IL-18, and TNF-α) as measured by qRT-PCR (n=4). (F) mRNA expression of pro-chemokine cytokines (MCP-1, MIP-1α, MIP-1β, and CX3CL1) as measured by qRT-PCR (n=4). (G) mRNA expression of ICAM-1 and VCAM-1 in aortas as measured by qRT-PCR (n=4). (H) Representative immunoblots for NLRP3 proteins in SCAP^+/+^ mice and SCAP^D443N^ mice (n=3). (I) Representative images of immuno?uorescence staining of NLRP3 proteins in the aortas of SCAP^D443N^ mice and SCAP^+/+^ mice (n=5). The data are presented as the means±SD of 3-5 independent experiments. *P<0.05, vs. the SCAP^+/+^ group. Statistical significance was calculated for the biological replicates by 2-tailed Student’s t test.

**Figure 6.**
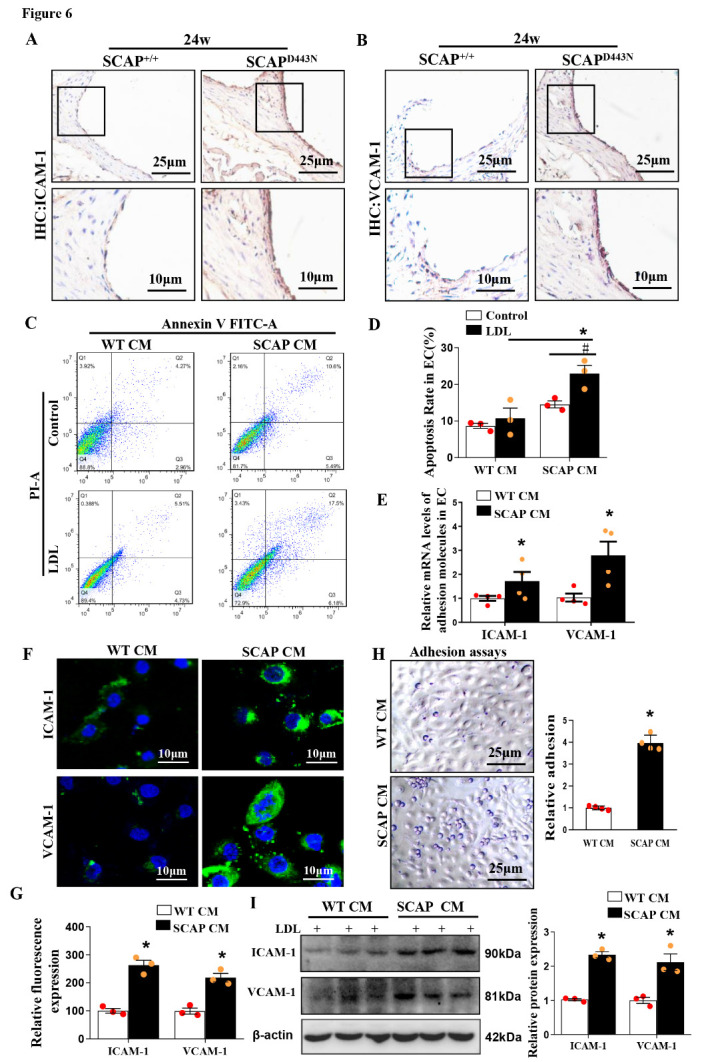
VSMCs overexpressing sterol-resistant SCAP promotes endothelial dysfunction by inducing inflammation. SCAP-overexpressing VSMC-conditioned media (SCAP CM) were collected from SCAP-overexpressing VSMCs. (A and B) Expression of ICAM-1 and VCAM-1 in aortas as determined by immunohistochemical staining. (C and D) Flow cytometry analysis of endothelial cell apoptosis after exposure to WT CM or SCAP CM with 100 μg/ml of LDL for 24 h, and apoptosis in endothelial cells was quantified. (E) The mRNA expression of ICAM-1 and VCAM-1 in endothelial cells after exposure to WT CM or SCAP CM with 100 μg/ml of LDL for 24 h was measured by qRT-PCR. (F and G) Representative images of immuno?uorescence staining of ICAM-1 and VCAM-1 proteins in endothelial cells after exposure to WT CM or SCAP CM with 100 μg/ml of LDL for 24 h. (H) Representative images of THP1 monocytes (small, round cells) adhered to endothelial cells (cobblestone shape) after exposure to WT CM or SCAP CM with 100 μg/ml of LDL for 24 h. (I) Expression of ICAM-1 and VCAM-1 protein in endothelial cells after exposure to WT CM or SCAP CM with LDL for 24 h as measured by Western blotting. The data are presented as the means±SD of 3 independent experiments. *P<0.05, vs. the WT CM+LDL group, ^#^*P*<0.05, vs. the SCAP CM group. Statistical significance was calculated for the biological replicates by 2-tailed Student’s t test.

### Sterol-resistant SCAP overexpression in VSMCs causes inflammation and lipid deposition in the aortas of SCAP^D443N^ mice

We found that lipid deposition in the aortic root sections of SCAP^D443N^ mice was significantly larger than that in control mice, regardless of whether they were fed a Western diet for 12 or 24 weeks (Fig. 5A-C). Next, inflammatory cytokines and serum lipid levels were examined. The plasma levels of TC, TG, LDL cholesterol, or HDL cholesterol show no significant changes between SCAP^D443N^ mice and control mice after 12 or 24 weeks of Western diet feeding (Fig. 5D). However, sterol-resistant SCAP overexpression significantly increased the mRNA expression of proin?ammatory cytokines (TNF-α, IL-1β, IL-6, and IL-18), chemokines (MCP-1, MIP-1α, MIP-1β, and CX3CL1), and adhesion molecules (VCAM-1 and ICAM-1) in the aorta compared with that in control mice (Fig. 5E-G). Western blotting analyses showed that sterol-resistant SCAP enhanced the protein expression of NLRP3 in the aortas of SCAP^D443N^ mice (Figure 5H). Immuno?uorescence staining revealed that the expression of NLRP3 was markedly increased in the aortas of SCAP^D443N^ mice (Fig. 5I). These results indicated that lipid deposition, aortic inflammation, and NLRP3 production were increased in the aortas of SCAP^D443N^ mice.

**Figure 7.**
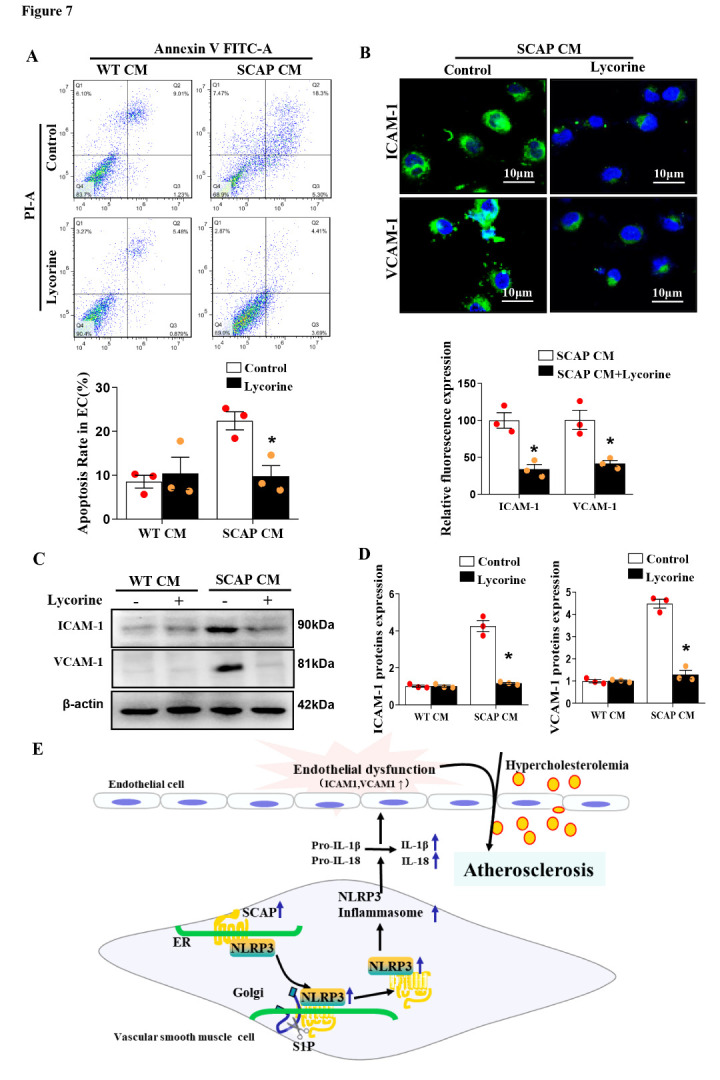
Inhibition of SCAP translocation ameliorates endothelial dysfunction by reducing NLRP3 in?ammasome activation. SCAP CM were collected from SCAP-overexpressing VSMCs after 4 h of treatment with or without lycorine. HUVECs were grown to confluence, and then HUVECs were treated with SCAP CM and 100 μg/ml of LDL for 24 h. (A) Flow cytometry analysis of endothelial cell apoptosis. (B) Representative images of immuno?uorescence staining of ICAM-1 and VCAM-1 proteins in endothelial cells. (C) Expression of ICAM-1 and VCAM-1 protein in endothelial cells as measured by Western blotting. (D) Quantification of ICAM-1 and VCAM-1 protein in endothelial cells in (C). (E) SCAP overexpression in VSMCs enhances local inflammation in the aorta via upregulation of NLRP3 expression and synergizes with hypercholesterolemia to promote endothelial dysfunction, which is implicated in atherosclerotic lesion initiation and progression. The data are presented as the means±SD of 3 independent experiments. *P<0.05, vs. the SCAP CM+LDL group. The data were tested using two-way analysis of variance, followed by Q-tests.

### Inflammation from sterol-resistant SCAP-overexpressing VSMCs induces endothelial injury

Interestingly, a strong tendency was observed toward an increased abundance of VCAM-1 and ICAM-1 in the aortas of SCAP^D443N^ mice after 24 weeks of Western diet (Figure 6A-B). ICAM-1 and VCAM-1 have been identified as markers of endothelial injury in mice. Here, we sought to explore the indirect effect of sterol-resistant SCAP overexpression in VSMCs on endothelial cells *in vitro*. Gain-of-SCAP function studies were performed in VSMCs using SCAP lentivirus transfection. Sterol-resistant SCAP-overexpressing VSMC-conditioned media (SCAP CM) were collected and cocultured with endothelial cells. Flow cytometry analysis showed that sterol-resistant SCAP overexpression increased the apoptosis of endothelial cells after exposure to the SCAP CM group compared with the SCAP^+/+^ VSMC-conditioned media (WT CM) group (Figure 6C-D). Immunofluorescence, qRT-PCR and Western blotting also demonstrated that the expression of ICAM-1 and VCAM-1 in endothelial cells was markedly upregulated in the SCAP CM group compared with that in the WT CM group (Figure 6F-G, I). To examine the effect of SCAP CM on monocyte binding, we used THP-1 monocytes to investigate monocyte adhesion to cultured ECs. Compared with WT CM treated ECs, SCAP CM led to an increase in endothelial cell-monocyte adhesion (Figure 6H). These data suggested that sterol-resistant SCAP overexpression in VSMCs induced endothelial cell injury.

### Inhibition of SCAP translocation attenuates NLRP3 in?ammasome activation and endothelial injury

To determine whether SCAP-induced NLRP3 in?ammasome activation in VSMCs is responsible for the induced endothelial injury, pharmacological inhibition of SCAP with lycorine was performed. According to flow cytometry analysis, lycorine abrogated the SCAP CM-induced apoptosis of endothelial cells (Figure 7A). This inhibitor also decreased the expression of VCAM-1 and ICAM-1 in endothelial cells (Figure 7B-D), suggesting that sterol-resistant SCAP induced NLRP3 in?ammasome activation in VSMCs, which was responsible for endothelial injury.

## DISCUSSION

In the present study, we generated a new mouse model of sterol-resistant SCAP (D443N mutation) in VSMCs crossed with the hyperlipidemic ApoE^-/-^ mouse to elucidate the mechanisms linking cholesterol metabolism and inflammatory disorders in the development of atherosclerosis. This mouse model allows us to investigate the role of SCAP-SREBPs in inflammatory signaling in the absence of cholesterol feedback regulation and revealed several novel and important findings. First, overexpression of sterol-resistant SCAP in VSMCs leads to an increase in atherosclerotic plaque formation independent of serum cholesterol levels; intriguingly, SCAP^D443N^ mice display hallmarks of fatty streak lesions characterized by loss of endothelial cell function and accelerated early atherogenesis. Second, the accelerated atherosclerosis in SCAP^D443N^ mice is largely attributed to increased local vascular inflammation, which is mediated by activation of the NLRP3 inflammasome. Finally, sterol-resistant SCAP overexpression leads to VSMC-derived inflammation and promotes endothelial injury through intercellular communication. Our study highlights the consequences of sterol-resistant SCAP overexpression in regulating local inflammation on vascular and endothelial cell function in atherosclerosis.

VSMCs are the major components of large and medium-sized arteries and are present at all stages of atherosclerotic plaque development [36]. Traditionally, the migration and proliferation of VSMCs in the intima contribute to atherosclerotic plaque formation [37]. At advanced stages, these cells form fibrous caps to stabilize vulnerable plaques [38]. The interplay between cholesterol homeostatic regulators and in?ammation in VSMCs was our research focus. Our previous studies showed that VSMC-specific SCAP knockdown prevented the development of atherosclerosis in ApoE^-/-^ mice by activating autophagy [39], with a decreased vascular inflammatory response. The present study extended these observations to show that mice overexpressing sterol-resistant SCAP in VSMCs clearly amplified hypercholesterolemia-induced atherosclerosis compared with ApoE^-/-^ mice, and the in?ammatory cytokines, chemokines and adhesion molecules significantly increased in the aorta. Based on our data, we believe that an inflammatory pathway is an important mechanism by which sterol-resistant SCAP in VSMCs amplifies atherosclerosis.

In human VSMCs, inflammatory factors disrupt LDL receptor feedback regulation, increase peripheral blood cholesterol uptake, and then form foam cells to promote the formation of atherosclerosis [40, 41]. The crystalline form of cholesterol that is present in early atherosclerotic lesions can induce inflammasome activation [27]. Indeed, recent evidence has shown that the SCAP/SREBP2 complex integrates NLRP3 inflammasome activation and cholesterol biosynthetic signaling during inflammation in proinflammatory macrophages [35]. The NLRP3 inflammasome has been reported as a central regulator of inflammation during the pathogenesis of atherosclerosis [42, 43]. IL-1β and IL-18 are two key in?ammatory factors in atherosclerosis development and are mainly regulated by the NLRP3 inflammasome [44]. Consistent with previous reports, our data indicated that sterol-resistant SCAP activated NLRP3 inflammasome signaling and increased inflammatory cytokine secretion in VSMCs. Importantly, the SCAP inhibitor lycorine significantly inhibited NLRP3 in?ammasome activation and ameliorated inflammatory cytokine secretion, indicating that SCAP is an important regulator of NLRP3 in?ammasome activation in VSMCs. Intriguingly, our results elucidate a novel mechanism of SCAP that can mediate communication between VSMCs and ECs in an NLRP3 inflammasome-dependent manner. After incubation with SCAP CM, we found that IL-1β and IL-18 secreted by VSMCs in?uenced the responses of ECs to a lipid stimulus, promoted endothelial injury, and increased the expression of adhesion molecules. Overall, we determined that, in addition to what has been observed for endothelial cells [24] and macrophages [35, 45], SCAP also regulates the activation of the NLRP3 inflammasome in VSMCs, thus inducing endothelial dysfunction and enhanced atherosclerotic progression.

Surprisingly, increased SCAP signaling not only promoted the development of advanced lesions but also enhanced the formation of new plaques at the thoracic aorta. Atherosclerotic lesions have traditionally been considered an “inside-out” response based on the core concepts that the inflammatory response initiates at intimal endothelial cells [46]. However, increasing evidence supports the new concept of an “outside-in” hypothesis in which the inflammatory response is initiated in the vascular adventitia and progresses to the intima endothelium, potentially inducing the formation of atherosclerotic lesions [47, 48]. In the setting of hyperlipidemia, PDGFRβ signaling promotes new plaque formation in the subendothelium of the thoracic aorta and is closely associated with inflammation of the adventitia and media [49]. Therefore, regardless of whether the “inside-out” hypothesis or “outside-in” hypothesis is more convincing, inflammation beginning in VSMC media might help initiate plaques at atherosclerosis-prone sites. We found that sterol-resistant SCAP significantly increased the expression of adhesion molecules in the intima endothelium that serve as markers of vascular inflammation and endothelial damage, thereby triggering lipid deposition at early stages of atherosclerotic plaque formation in SCAP^D443N^ mice. Endothelial dysfunction is generally regarded as a crucial and initial step in atherosclerotic lesion development and progression [50]. Inflammatory cytokines are secreted by almost all cell types involved in the formation and progression of atherosclerosis, exerting multiple crosstalk effects among endothelial, macrophage, and smooth muscle cells. Activated VSMCs can release a series of chemokines and cytokines and interact with infiltrating immune cells to propagate and amplify vascular inflammation and atherosclerosis [51, 52]. VSMCs differentiate into lymphoid tissue organizer-like cells and secrete lymphorganogenic chemokines that promote inflammation of the aorta adventitia [49, 53]. VSMCs interact with ECs and provide a synergistic effect to promote the progression of atherosclerosis [49]. The literature describing endothelial dysfunction is immense, much of which is focused on the effects on VSMCs. However, there is growing evidence that the relationship between ECs and VSMCs is not a simple one-way interaction from the endothelium to VSMCs in atherogenesis; rather, changes that occur in VSMCs have regulatory effects on endothelial function. Increased mechanical stretching leads to VSMC-derived microparticle production, which contributes to EC dysfunction and inflammation, leading to thoracic aortic aneurysm [54]. Exosomes secreted from VSMCs can transfer miR-155 to endothelial cells, which induce increased endothelial permeability and enhance atherosclerotic progression [55]. Combined with our results, inflammatory cytokines released by media VSMCs likely work together to modify the aorta and may induce new niches for atherosclerotic plaque initiation.

In summary, our data indicated that SM22 promoter-driven sterol-resistant SCAP in VSMCs promotes the secretion of inflammatory factors via the upregulation of NLRP3 expression, which subsequently leads to endothelial injury and ultimately accelerates the occurrence and development of atherosclerosis. Given the importance of SCAP in regulating NLRP3 inflammasome activation, developing effective inhibitors targeting SCAP may provide effective strategies for the prevention and treatment of chronic metabolic diseases.

## Supplementary Materials

The Supplemenantry data can be found online at: www.aginganddisease.org/EN/10.14336/AD.2020.1120.


