# Supplementary material for: Sterol-resistant SCAP Overexpression in Vascular Smooth Muscle Cells Accelerates Atherosclerosis by Increasing Local Vascular Inflammation through Activation of the NLRP3 Inflammasome in Mice
Source: Aging Dis. 2021 Jun 1;12(3):747–63. doi: 10.14336/AD.2020.1120 (PMC8139202; doi:10.14336/AD.2020.1120)
Supplement: Supplementary file 1 [file AD-12-3-747-s.pdf]

SUPPLEMENTARY DATA

Sterol-resistant SCAP Overexpression in Vascular Smooth Muscle Cells Accelerates Atherosclerosis by Increasing Local Vascular Inflammation through Activation of the NLRP3 Inflammasome in Mice

Danyang Li[1,#], Mihua Liu[1,#], Zhe Li[1], Guo Zheng[1], Amei Chen[1], Lei Zhao[1], Ping Yang[1], Li Wei[1], Yaxi Chen[1*], Xiong Z. Ruan[1,2.3*]

© 2020. Li D et al. Published online at http://www.aginganddisease.org/EN/10.14336/AD.2020.1120

SUPPLEMENTARY DATA

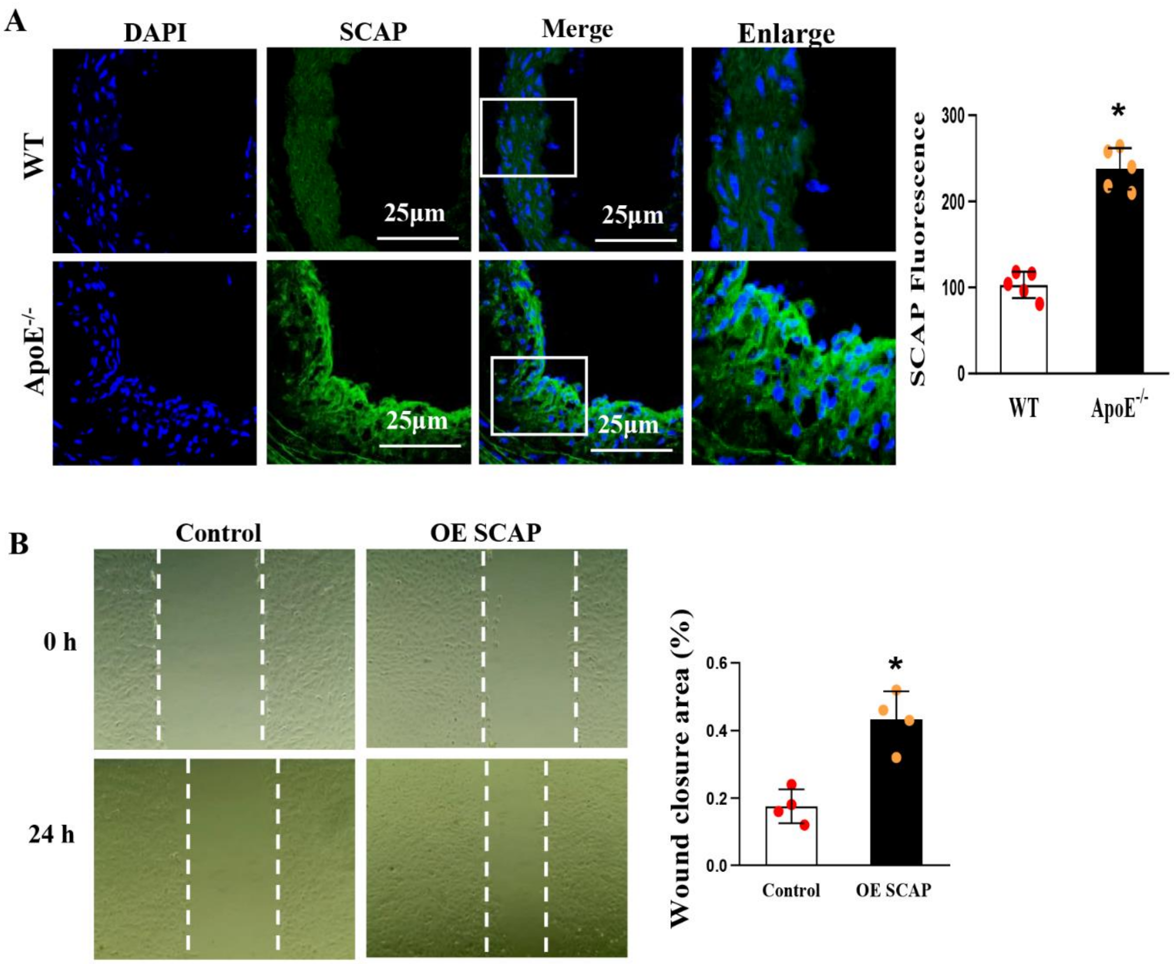

Supplementary Figure 1. The expression of SCAP is increased in atherosclerotic lesions, and SCAP induces VSMC migration. (A) Representative images of immunofluorescence staining of SCAP in the atherosclerotic lesions of WT and $ApoE^{-/-}$ mice after 12 weeks of Western diet feeding. (B) Cell migration after SCAP overexpression in VSMCs was analyzed by wound healing assays. The data are presented as the means±SD of 3 independent experiments. *P<0.05, vs. the WT group or control group. Statistical significance was calculated for the biological replicates by 2-tailed Student's t test.

© 2020. Li D et al. Published online at http://www.aginganddisease.org/EN/10.14336/AD.2020.1120
